# BART Streams: Real-time Reconstruction Using a Modular Framework for Pipeline Processing

**Published:** 2025-12-02

**Authors:** Philip Schaten, Moritz Blumenthal, Bernhard Rapp, Christina Unterberg-Buchwald, Martin Uecker

**Affiliations:** 1 Institute of Biomedical Imaging, Graz University of Technology, Graz, Austria; 2 German Centre for Cardiovascular Research (DZHK), partner site Lower Saxony, Germany; 3 Institute for Diagnostic and Interventional Radiology, University Medical Center Göttingen, Germany; 4 Clinic for Cardiology and Pneumology, University Medical Center Göttingen, Germany; 5 BioTechMed-Graz, Graz, Austria

**Keywords:** MRI, Streaming, Real-Time MRI, Real-Time Reconstruction, Interventional MRI

## Abstract

**Purpose:**

To create modular solutions for interactive real-time MRI using reconstruction algorithms implemented in BART.

**Methods:**

A new protocol for streaming of multidimensional arrays is presented and integrated into BART. The new functionality is demonstrated using examples for interactive real-time MRI based on radial FLASH, where iterative reconstruction is combined with advanced features such as dynamic coil compression and gradient-delay correction. We analyze the latency of the reconstruction and measure end-to-end latency of the full imaging process.

**Results:**

Reconstruction pipelines with iterative reconstruction and advanced functionality can be built in a modular way using scripting. Latency measurements demonstrate latency sufficient for interactive real-time MRI.

**Conclusion:**

With the new streaming capabilities, real-time reconstruction pipelines can be assembled using BART in a flexible way, enabling rapid prototyping of advanced applications such as interactive real-time MRI.

## 1 Introduction

Real-Time MRI (RT-MRI), the process of simultaneous measurement, reconstruction and low-latency display of MR images [1] provides a “live stream” from the inside of the human body. It is an important tool with emerging clinical applications, such as interventional [2–6] or fetal MRI [7].

RT-MRI usually requires high frame rates, e.g. to resolve cardiac motion [8], speech production [9, 10], or joint motion [11]. It follows that relatively little data is available to reconstruct each frame. While this can be addressed by the use of advanced iterative reconstruction algorithms, RT-MRI presents two additional challenges: First, algorithms for (interactive) RT-MRI must be causal in the sense that reconstructed frames can only depend on previously acquired data, not on the whole dataset. Second, as latency must be kept low, reconstruction must be fast.

One method which is well-suited for RT-MRI is the real-time regularized non-linear inversion (RT-NLINV) algorithm [12, 13]. Based on classic regularized non-linear inversion (NLINV) [14], RT-NLINV offers particularly high undersampling factors and is causal in the aforementioned sense. As iterative reconstruction is computationally expensive, running iterative RT-MRI reconstruction algorithms such as RT-NLINV in real-time typically requires optimized software and hardware acceleration e.g. through the use of Graphical Processing Units (GPU) [15–17].

The BART toolbox for computational MRI [18] is a comprehensive framework which provides a wide range of reconstruction, calibration [19], machine-learning [20, 21], and signal processing tools. Its optimized code and its existing support for GPU computing make it an ideal choice for the development of real-time reconstruction methods. Another core feature of BART is its modular design as a toolbox: Algorithms are made available as individual command line tools, which typically take several parameters as well as in- and output data file names, enabling the construction of powerful reconstruction pipeline by combining the different tools in a script.

High-quality RT-MRI requires not only highly optimized reconstruction, but also the ability to stream the data and reconstructed images during the measurement. Each reconstruction tool used in the reconstruction pipeline needs to perform its task as soon as a raw-data frame is available, as opposed to waiting for a complete dataset.

In this work, we integrate new stream processing capabilities into BART. The new functionality facilitates building reconstruction pipelines suitable for RT-MRI with existing BART tools. Importantly, our approach to streaming builds on standard Unix system utilities, which allows us to preserve the full modularity of BART. This is demonstrated using examples for interactive real-time MRI based on radial FLASH, where iterative reconstruction is combined with advanced features such as dynamic coil compression and gradient-delay correction. We analyze the latency of the reconstruction itself and measure the end-to-end latency of the complete imaging process.

## 2 Methods

We will first give an overview over the architecture of BART, then discuss multi-dimensional arrays, and then explain the new support for looping, and streaming, which is then used for real-time processing.

### 2.1 BART Structure

BART is a modular reconstruction framework which enables building complex reconstruction algorithms through combination of various individual components. On the lowest level, a numeric library provides generic functions for operations on multidimensional arrays (md-arrays). Many of these operations can be accelerated using GPUs, or with the Message Passing Interface (MPI) [22] toolkit, enabling distributed computing on scientific high-performance clusters and multi-GPU usage [23]. The multi-dimensional operations are complemented by an operator library, which provides a simple way of constructing operators, e.g. the forward operator describing an MRI measurement, while automatically providing its adjoint in the case of a linear operator, or the derivative for non-linear operators.

While the code is organized in the form of reusable libraries, their direct use is rarely needed. For most use cases it is sufficient to interact with BART using its command line interface, which consists of a set of high-level tools operating on md-arrays. All commands take several individual options and typically input and output file names. A single driver command, bart, acts as the unified entry point to BARTs command line interface. It can be called directly from a command line or from scripting languages such as BASH, Python or Matlab. The bart call is followed by a set of general options and the name of the tool which should be run. There are basic tools such as fft which calculates the Fourier transform of an md-array, as well as many commands for MRI-specific tasks such as whitening (whiten), coil compression (cc), calibration of sensitivities (ecalib, ncalib), parallel imaging and compressed sensing (pics), model-based reconstruction (moba), machine-learning reconstruction (reconet, nlinvnet), and much more.

The tools act as building blocks and can be combined in a modular way to construct advanced reconstruction pipelines for all kinds of MRI applications. A complete reconstruction implemented with BART thus typically consists of a script that calls various BART commands that operate on md-arrays.

For real-time reconstruction, it must be possible to split up, i.e. slice, the multidimensional input space and process each subset of the data as it becomes available. In other words, the reconstruction script has to be organized as a pipeline. To support this in BART while preserving its modular character, the tools were enhanced to support streaming of slices of md-arrays.

### 2.2 Slicing

Suppose for n∈ℕ that we want to split up an n-dimensional array $X\in {\mathbb{C}}^{N_{0}\times N_{1}\times \ldots \times N_{n-1}}$. In the following, the entries $X_{\left(i_{0},\ldots ,i_{n-1}\right)}$ of X are referenced using the multi-index

$$\left(i_{0},\ldots ,i_{n-1}\right),\;i_{j}\in {\mathbb{N}}_{0}\;\text{and}\;i_{j}<N_{j}-1.$$

**Figure 1:**
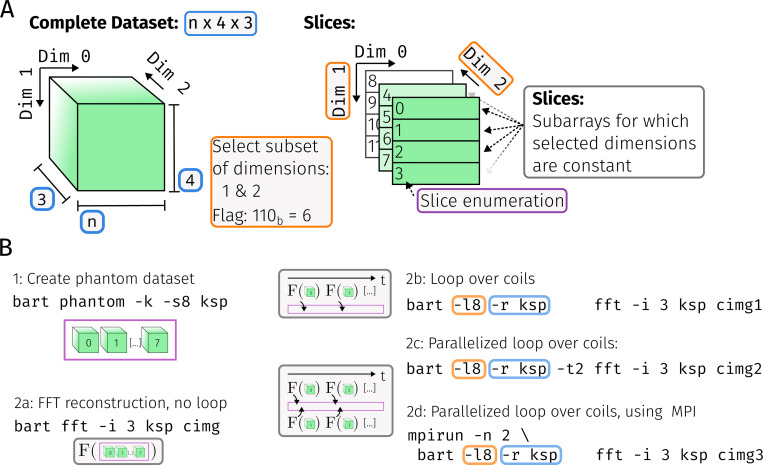
**A**: Schematic of how a three-dimensional array can be sliced. The cube on the left symbolizes the full three-dimensional array. By selecting a set of dimensions {2,1} ⊂ {2,1,0}, the array can be decomposed into a set of slices for which the index along dimension 0 stays constant, as shown on the right. The set of selected axes can be efficiently encoded as a bitmask 6 = 1 × 2^2^ + 1 × 2^1^ + 0 × 2^0^, and every slice is uniquely identified through a serial number. **B**: Example command lines which demonstrate how slicing can be used in BART. First, a k-space dataset with multiple channels is generated using command 1. Commands 2a-d then calculate the FFT of it by using the fft-tool in different ways: Command 2a processes the dataset in a single invocation (*combined*). Command 2b processes all channels serially. Command 2c uses the -t (threads) option to process all channels in parallel in two threads. Note that 2a will typically use multiple threads as well, but on a lower level within the FFT implementation itself. Finally, 2d uses mpirun to process the dataset on two nodes [22] or two GPUs.

One method to subdivide this array is slicing i.e. to select a set of axes, and take those subsets of the array within which the indices of the selected axes stay constant. This concept is illustrated in Figure 1A.

To give a precise description, if A:={0,1,…,n−1} is the set of all axes of the array, then, for some m<n, m∈ℕ, a subset of m axes S⊂A is first selected for slicing. Any given multi-index can then be decomposed into two multi-indices a and b:

(1) $$\left(i_{S(0)},\ldots ,i_{S(m-1)}\right)=\left(a_{0},\ldots ,a_{m-1}\right),$$

(2) $$\left(i_{F(0)},\ldots ,i_{F(n-m-1)}\right)=\left(b_{0},\ldots ,b_{n-m-1}\right),$$

where F=A\S is the set of free axes, and S(k) and F(k) denote the k-th smallest entry of the set S and F, respectively. In other words, X is split into a number of n−m dimensional sub-arrays or “slices” by fixing part of the multi-index in every slice.

Every slice is associated with one specific multi-index a. The set of all slices can be enumerated by flattening the multi-index a into a serial number, i.e. a single integer

(3) $$c=\sum_{k=0}^{m-1}a_{k}\left(\prod_{l=0}^{k-1}N_{S(l)}\right).$$

As common in BART, the selected set of dimensions S used for this slicing operation can be efficiently encoded as a single integer using a bitmask. This is done by interpreting the pattern of selected/non-selected axes as a binary number

(4) $$\text{bitmask}(S)=\sum_{i\in S}2^{i}.$$

### 2.3 Looping

The decomposition of md-arrays into slices is used by BART in two different but related contexts, i.e. streaming and looping. The idea of looping is to apply the functionality of a BART tool sequentially to every slice, instead of applying it once to the whole array (normal/*combined* operation). As the BART tool which is invoked then does not “see” the entire array, but instead operates on a single slice, this can sometime lead to semantic differences, e.g. when a full 3D reconstruction is numerically different to reconstruction decoupled into slices. For many BART commands, exactly the same result is computed when the computation is uncoupled along the loop dimensions, but the memory requirements will typically be much smaller for looped operations, because only memory for computation on one slice is required. Also, because the computation is now done independently for each slice, it can be distributed across different nodes of a high-performance compute cluster or across different GPUs.

The looping functionality in BART is controlled using a newly added set of options, referred to as *loop options*. Using the -l (loop) flag, a set of slice dimensions can be passed to the bart driver command as a bitmask (see Equation (4)). Furthermore, the dimensions of the full md-array need to be passed, either by specifying a reference file using the -r (reference) flag, or explicitly by specifying the size of each selected dimension as an argument in the -e (end) flag. Figure 1B illustrates the loop options in a self-contained example.

Extending upon the previously introduced decomposition, subsets of an array can be obtained by selecting only some of its slices. This is done with the -s (start) and -e (end) flags, which can be used to select only a specific range of slices. The tool is then called repeatedly until all slices in the selected range have been processed. Together, the loop options enable access to subsets (slices) of an array, resembling how BART works with md-arrays internally, and similar to array-views used in other numerical packages and programming languages such as Fortran [24] or NumPy [25].

### 2.4 Streaming

The goal of BART’s streaming feature is to enable pipeline processing by letting the tools send and receive slices of md-arrays during the computation. This enables a chain of tools to simultaneously process different slices as part of a single reconstruction pipeline. Output is then produced as soon as the first slice was processed by all pipeline stages.

**Figure 2:**
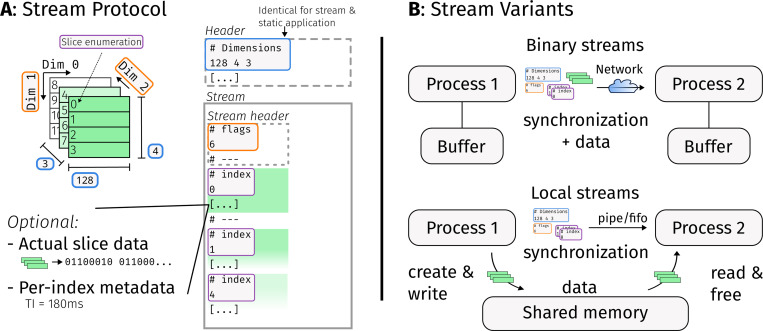
Structure of the BART stream protocol. In part **A**, the sliced array from Figure 1 is shown on the top left. A BART process can communicate this array in a slice-wise fashion by first transmitting a header, which contains the dimensionality of the array, shared memory information (if applicable) and optional metadata such as BART version. The format is identical to the static header of BARTs native .cfl/.hdr file format. The header is followed by a stream header which includes a flag describing the dimensions that are used for slicing. Then, the individual slice indices are transmitted once they have been processed. Slice indices may be followed by actual slice data in binary form, and per-slice metadata. Part **B** shows two transport mechanisms used with the stream protocol. Metadata, synchronization and actual data can be sent together through the same channel, permitting to use the protocol over the network (top), referred to as the binary version of the BART stream protocol. Alternatively, if both processes have access to shared memory, this can be used to share the actual array data (bottom). This is the default behavior when sending data from a BART command, unless the --stream-bin-out option is used.

Streaming requires communication between different processes and thus, a protocol. Hence, we implemented a simple streaming protocol, illustrated in Figure 2. It requires a reliable byte stream and optionally employs shared memory, but does not otherwise depend on the underlying communication mechanism. Actual array data is either transferred inline or made available through shared memory.

The streaming protocol can be seen as a new file format that extends BART’s native cfl-format, and BART will use streaming automatically in two cases based on the file name of the argument.

First, streaming is active if a hyphen is specified as file name, which by convention is understood as a reference to standard input/output. BART stream output can be printed to the screen in this way, which is useful for debugging. Typically though the goal is to connect multiple BART commands, which can be achieved by using pipes. Originally introduced with UNIX in 1973 [26], they are nowadays an integral part of any Linux-based distribution and also available on most other operating systems. The vertical-bar or pipe operator | is used in common shells on UNIX-derived systems (see [26]) to start two processes in parallel, such that the standard output of one is connected to the standard input of the other by means of a pipe.

The second case in which BART uses the streaming protocol is when a file name ends in .fifo. A *fifo* (First-In, First-Out) file, or named pipe, is a pipe which is associated with a path in the file system. If the *fifo* file does not yet exist, BART will create it. *Fifo*s enable streaming of multiple in-/outputs at the same time, which is needed to write complex reconstruction scripts with streaming support, as described later in Section 2.5.

By using the option --stream-bin-out, the binary version of the protocol is enabled, which forces inlining of the data and avoids relying on shared memory. In this way a BART command can send data to another machine. For example, one can use the Transmission Control Protocol (TCP) [27], by sending BART output to, or receiving input from tools such as ncat [28].

Generally, all BART tools directly support streaming. Within BART, a set of functions ensures synchronization across processes. Most importantly, the stream_sync function is used on input streams to wait for input data or on output streams to notify subsequent processes when a certain slice has been completed.

On the level of BART tools, streaming is handled in three different ways. First, the default method is to wait (block) until all data in a stream has fully arrived. This is essentially the same behavior that would also occur if no streams were used.

A more powerful method is to combine streaming with the loop functionality. If a specified reference file is a stream, the stream flags are directly used as loop flags and the BART tool is invoked on every slice as soon as it is received. Thus, the combination of the loop functionality and the stream protocol allows any BART tool to be used as part of a pipeline without blocking it.

Some operations such as regularization along the time dimension make use of information from previously processed slices. To make such operations easier, several tools have been made stream-aware, which is the third way in which a BART tool might use streaming. These tools integrate synchronization of the streams with their internal logic using the stream_sync function. For example, this enables regularization onto and initialization with previous frames in real-time application of nlinv.

### 2.5 Applications

We demonstrate the application of BART streams in three different exemplary scenarios.

**Figure 3:**
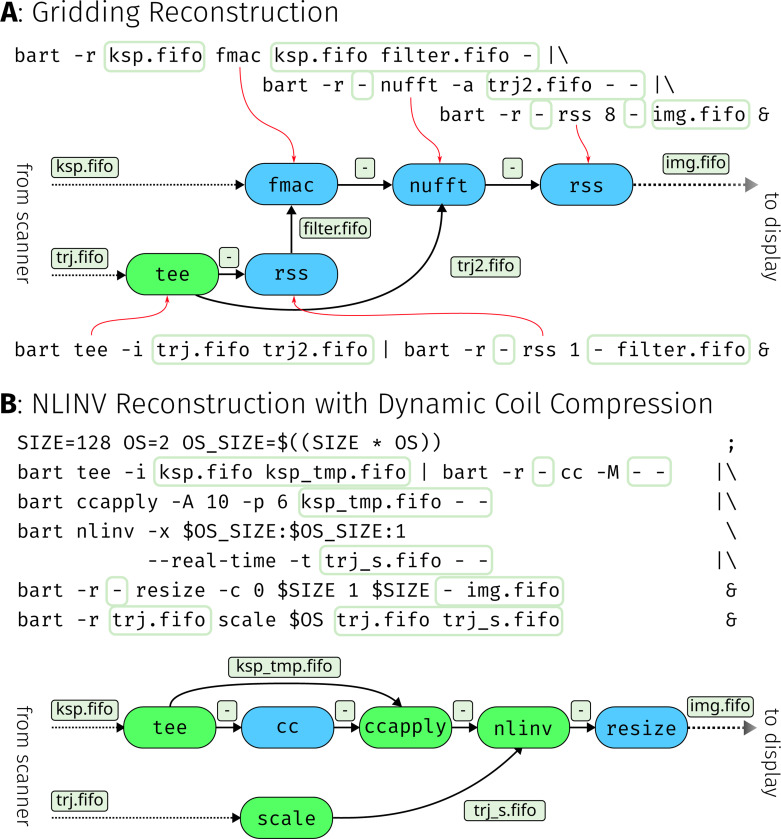
Part **A** shows a simple reconstruction pipeline using a Ram-Lak filter, an adjoint NUFFT, and root sum-of-squares coil combination. Shell code with which the pipeline can be created is shown along with a representation of the computational graph. Edges represent files, large colored nodes represent BART processes. ksp.fifo and trj.fifo are named pipes, which are expected to deliver incoming k-space data and trajectory from the scanner or previous processing steps. Similarly, img.fifo provides reconstructed images for further processing and display. Colors in the process nodes are used to highlight how tools can utilize streams in different ways. Blue commands use the loop options, while green commands are stream-aware. Part **B** shows an example for an advanced reconstruction where geometric coil compression and NLINV are used for real-time reconstruction of radial data. Incoming raw data is first split with the tee command and forwarded to two different tools, cc and ccapply. The cc tool calculates the coil compression matrices, but does not apply them. Instead, they are forwarded to the ccapply tool, which applies the received matrices to the incoming raw data and forwards the compressed k-space to the nlinv reconstruction. A subsequent resize command removes oversampling in the image domain.

To begin, reconstruction for RT-MRI using radial sampling is performed using the adjoint non-uniform FFT (NUFFT) or *gridding* [29]. Here, the raw k-space data from the scanner is first multiplied with the Ram-Lak filter [30], to compensate for non-uniform sampling density of radial sampling. Afterwards, the adjoint NUFFT is applied to obtain coil images, which are combined using root sum-of-squares. Compared to iterative reconstructions, this scheme is relatively simple and can run in real-time even without GPU acceleration. Figure 3A shows an RT-MRI pipeline implementation of this reconstruction using BART streams.

Practical reconstruction pipelines typically use more advanced reconstruction algorithms and many additional pre- and post processing steps. With interactive real-time MRI in mind, we will now consider a more complex example using NLINV and dynamic coil compression.

Coil compression is often employed to limit memory usage and improve reconstruction speed. It makes use of Singular Value Decomposition (SVD) to calculate a compression matrix, which is then applied to the raw data to reduce the set of physical coil signals to a smaller number of virtual coils [31]. In interactive MRI, it is important to update the compression matrix for each frame, because coil profiles can change due to slice repositioning. However, simply performing individual SVD-based coil compression of every frame causes artifacts. As the basis computed by the SVD is not unique, discontinuities in the profiles of the compressed virtual coils can occur, which can in turn produce flickering in the image series. To avoid this problem, we recently adapted geometric coil compression [32] to RT-MRI by performing alignment of the compression matrices along the time dimension [33]. Using this adjusted coil compression method requires incoming data to be split up, processed, and then recombined in real-time, which can now be accomplished using the streaming functionality of BART. Figure 3B shows a reconstruction pipeline based on NLINV with dynamic coil compression.

As a final example, a complete state-of-the-art reconstruction pipeline for real-time MRI was created. It takes the form of a BASH script which launches different reconstruction pipelines based on user-provided options. Furthermore, it includes preprocessing steps such as coil compression and gradient delay correction based on RING [35] as well as post processing steps such as a median filter and a non-local means filter [36]. The script can process data acquired either with radial turn-based trajectories (see [37]) or using the rational approximation of golden angles [38].

Reconstructions from three script configurations are compared in the results. The first setting uses the gridding-based reconstruction from Figure 3A, combined with a sliding window / view-sharing strategy on data acquired with a turn-based radial trajectory [37]. This example can run on lower-end hardware, as it uses only four virtual coils and omits steps such as gradient-delay correction.

The second example does not employ sliding-window, uses RT-NLINV for reconstruction, and includes gradient delay correction. It strikes a balance between high image quality and low latency.

**Figure 4:**
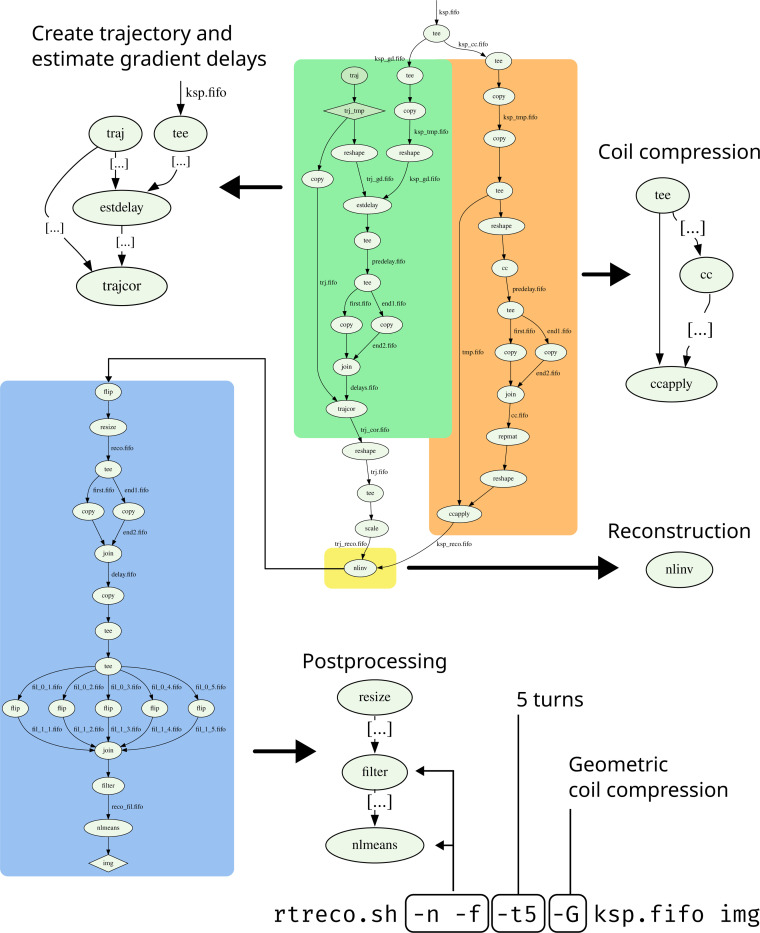
State-of-the-art reconstruction pipeline for real-time MRI. Different sections of the reconstruction pipeline are highlighted, and characteristic parts are also shown in a zoomed-in, abbreviated version next to the full graph. Round nodes represent BART invocations while edges between tool nodes represent pipes. The invocation of the real-time reconstruction script corresponding to this graph is shown at the bottom, along with an explanation of command line options. The figure is based on an auto-generated graph, which was created by tracing every BART tool invocation and translating this trace into a graph description language, and was rendered with dot [34].

The third configuration corresponds to the pipeline shown in Figure 4. By integrating geometric coil compression (cf. Figure 3B) and a frame-wise non-local means filter, as well as using a higher number of iterations in RT-NLINV, it achieves the highest image quality.

Furthermore, we analyse the impact of geometric coil compression and visualize how gradient delays change over the course of an RT-MRI measurement with interactively updated slice geometry. Using the gradient delay model from [35], gradient delays are written as symmetric 2×2 matrices, which, when applied to the readout direction vector, produce the shift in k-space that needs to be corrected. By eigendecomposition, these matrices can be visualized as ellipses whereby the principal axes of the ellipse correspond to the strength and direction of the gradient delay.

### 2.6 MRI Scans

We show exemplary results of the real-time reconstruction script on cardiac scans of a healthy volunteer, who was examined with approval by the local ethics board and after giving written informed consent. Measurements were done with a Magnetom Vida 3T (Siemens Healthineers, Erlangen, Germany) using a radial FLASH [39] sequence with random RF-spoiling [40] and a turn-based pattern with 5 turns and 13 spokes per turn [37]. Further parameters are given in Table 1. In addition, phantom scans were performed using the same sequence and a water-filled test tube that can be moved in the scanner.

In our setup, the real-time reconstruction is started on demand by a service manager (systemd). This avoids occupying GPU resources when the reconstruction is not running, but it also facilitates a possible integration into the cloud. Data is exchanged with the MRI scanner using the binary version of the BART streams protocol (Section 2.4) with a custom data import/export software that is integrated into the vendor software running on the scanner.

### 2.7 Latency and Performance Measurements

The computational performance of the new BART features was evaluated. In a setting with only a single compute node and a single thread, BARTs looping mode will typically be slower than normal/combined operation due to overhead such as repeated initialization. Hence, we first quantify the runtime overhead imposed by streaming/looping in this case. Furthermore, we measure the peak amount of memory which was simultaneously used by BART in the computer’s main memory (resident set size).

First, an md-array of complex random numbers $\text{x}\in {\mathbb{C}}^{n_{r}\times n_{p}\times n_{s}}$ is created, representing raw data from a Cartesian multi-slice MRI measurement for different matrix sizes $n_{r}$, $n_{p}$ with oversampling in readout direction $n_{r}=2n_{p}$ and a varying number of slices $n_{s}$.

An inverse 2D-FFT of every slice is then performed in three different ways (cf. Figure 1). First, with a regular BART command, resulting in combined processing of the data:

bart fft -i3 x out

Second, the 2D-FFT is sequentially performed on every slice using the loop options:

bart -14 -r x fft -i 3 x out

Third, data is read by the looped fft command from a previously created text file using the stream protocol:

bart -14 -r x copy x - > stream.txt
bart -l4 -r - fft -i 3 - out < stream.txt

In a second experiment, we measure the speed-up for a simple reconstruction pipeline where streaming and looping enable parallelization of consecutive reconstruction steps. For this, a customizable delay is added to the input stream, simulating per-slice acquisition time in an MRI measurement. The output of the copy-tool is then forwarded to the fft-tool directly via a pipe, causing the processes to run in parallel:

bart -14 -r x copy --delay <delay> x - |\
bart -14 -r - fft -i 3 - out

This is compared to the static variant:

bart -14 -r x copy --delay <delay> x - |\
bart fft -i 3 - out

where the reconstruction can only begin after the copy-process has processed all slices. The experiment is repeated for different delays between 0ms and 130ms.

Wall-clock run time is measured using the date program from GNU Coreutils. The experiments were repeated 25 times to measure standard deviation across runs, and performed on a standard workstation using an Intel Xeon W-2123 CPU running at 3.60GHz.

To assess latency for real-time MRI, phantom measurements we performed. We use the real-time reconstruction script as described in Section 2.5, with RT-NLINV and gradient delay correction, but without NLM-filter and without geometric coil compression. The reconstruction was accelerated using an Nvidia H100 GPU (80 GB HBM3, SXM) on a system with an AMD EPYC 9334 CPU.

**Figure 5:**
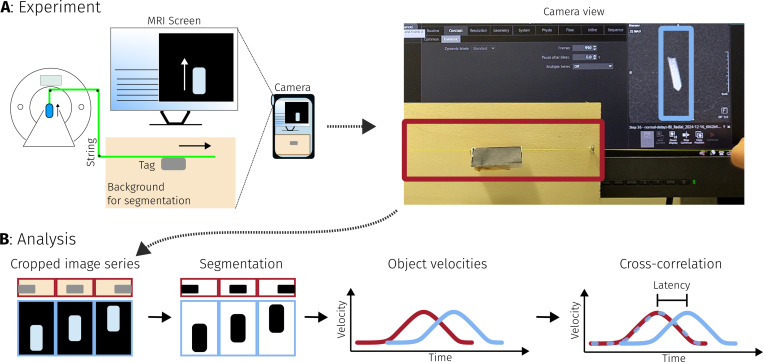
Part **A**: Schematic overview of the end-to-end latency measurement experiment setup. A water-filled test-tube (blue) inside the MRI scanner is pulled up from the control room using a long string (yellow). Images from the real-time sequence are shown on the MRI console screen. A smartphone camera records movement on the MRI screen and movement of a paper tag (gray) attached to the string. A single frame from the resulting video is shown on the top right. Part **B** illustrates how the resulting video is processed: The video is segmented, and the obtained object velocity curves are cross-correlated to obtain the delay between both movements.

Apart from acquisition and the reconstruction, several other factors such as network transfer times, filters used in post processing, and image display are expected to contribute to the total latency. Therefore, we determined several different latencies:

- Latency in BART is calculated as time difference between arrival of the last spoke and transmission of the reconstructed frame. Preprocessing, reconstruction and post processing were also measured individually. Timestamps are recorded using the gettimeofday function from glibc.
- Latency BART + Network as seen from the software running on the scanner is measured by taking timestamps when the last spoke is sent over the network and after the corresponding frame has been received. This also includes data transfer times and network delays.
- End-to-end latency, i.e. the time between an event happening in the scanner until it is shown on the MRI screen, is measured using an experimental setup as described in [41] and illustrated in Figure 5. Movies were recorded using a smartphone camera (Fairphone 4, Fairphone B.V., Amsterdam).

Because the MRI acquisition starts as soon as possible without waiting for the BART reconstruction pipeline to be initialized, there is typically a small initial delay when the reconstruction pipeline is set up. This is followed by a brief period of time in which the reconstruction catches up with the acquisition and the latency decreases towards a steady state, which is reported here as *steady state latency*.

## 3 Results

### 3.1 Performance

#### 3.1.1 Numerical experiments

We first present results on the performance of the two main additions to the BART software, streaming and looping.

**Figure 6:**
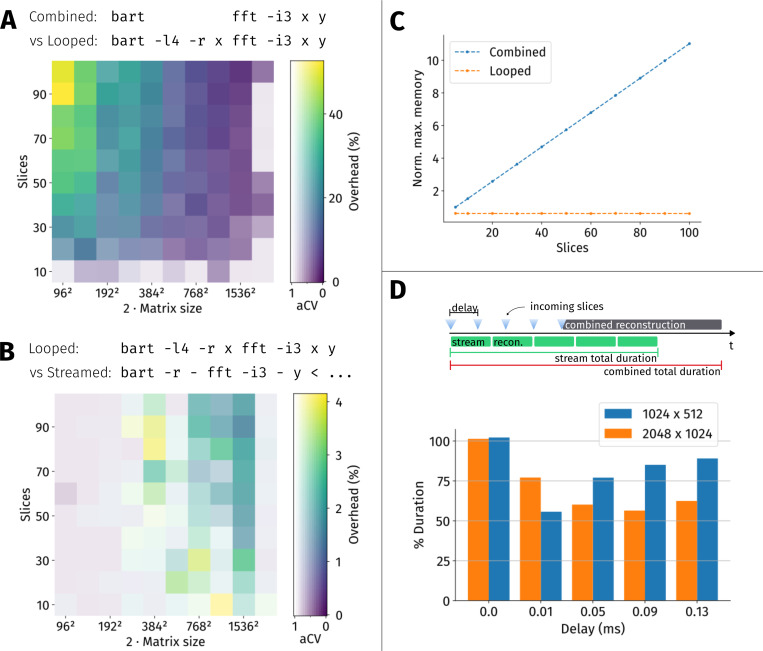
The performance of BART looping and streaming is shown here by visualizing the runtime of an inverse FFT on arrays of varying size. Part **A** shows a heat map of additional time required when performing a looped FFT relative to the normal/combined FFT, for different problem sizes. The opacity/lightness is based on an adjusted coefficient of variation (aCV): Fully saturated colors represent a distinct overhead, while light colors indicate that the average run-to-run difference is at least as big as the calculated overhead. Part **B** compares the overhead of streaming relative to looping, similar to part A. Part **C** visualizes the memory advantage of looped versus combined operation for a matrix size of 512 × 256 with varying number of slices. Memory usage is normalized to the amount required for a single slice, i.e. array size 512 × 256 × 1. Part **D** shows the speed-up due to reduced waiting times in pipeline operation, i.e. when the process producing the data (acquisition) and the reconstruction can be parallelized.

As described in Section 2.7, looping and streaming can under some conditions create computational overhead. Figure 6A shows the overhead when performing a looped FFT compared to normal FFT. For small arrays with many iterations / slices, there is a large relative overhead. For example, the FFT of a 96×48 array with 100 slices takes roughly 50% more time when using looping over the slices. However, this overhead is actually small in absolute units due to the small problem size. In this case, the extra time spent due to looping is 10(1)ms.

The relative overhead gets small for larger problem sizes. For example, albeit the looped FFT of a 2048 by 1024 array in 100 slices is 400(150)ms slower, this amounts to an only 2% longer time, and the average run-to-run deviation in computation time has the same order of magnitude. Overhead thus becomes negligible compared to the overall runtime of the process, as most of the time is spent with computation. For few slices or large problem sizes, the differences between looping and combined operation are small relative to the variation over repetitions.

In Figure 6B, we repeat this analysis for the streaming protocol (Section 2.4), comparing the extra time required when performing looping with a streamed input as opposed to looping alone. The maximum overhead is several times smaller than in the previous analysis. Furthermore, the overhead induced by the stream protocol is smaller than the average run-to-run variation in most cases. Also, there is no clear trend towards any greater or smaller overhead depending on the matrix size.

One advantage of looping is that it can help to reduce memory requirements. As illustrated in Figure 6B, with looping, peak memory usage is independent of the number of slices while it grows linearly with normal i.e. combined operation.

Furthermore, combined streaming and looping enables parallelization of successive data processing steps, and thus can speed up computation. This is illustrated in Figure 6D. Depending on matrix size and delay (slice acquisition time), total elapsed time was reduced down to a minimum of 56% of the reference.

#### 3.1.2 End-to-End Latency

**Figure 7:**
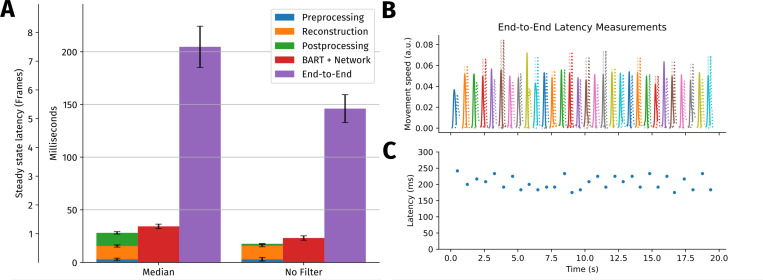
Latency measurements for RT-MRI. Part **A** visualizes results from three different latency measurement methods described previously. Latency determined within BART is further separated into three different steps: preprocessing, reconstruction and post processing. The times are shown for two measurements, with and without a five-frame median filter in the post processing chain. Error bars indicate standard deviation over the course of the time series. An initially higher end-to-end latency in the first few seconds is excluded for calculating the steady-state end-to-end latency shown here. Part **B** and **C** on the right highlight details of the end-to-end latency measurement. In **B**, the different movement curves from pulling on the string can be seen. Solid lines are calculated from the optically observed movement, while dashed lines correspond to the movement observed in the reconstructed images shown on the MRI console. The optimally shifted MRI movement curves are shown with transparent dashed lines. In **C**, the resulting time series of end-to-end latency measurements is shown.

The latency of our RT-MRI setup was measured using different methods (Section 2.7). The measurement was repeated three times, with almost identical outcome. One instance of these measurements is shown in Figure 7.

The BART-internal timing measurements reveal the amounts of time required for preprocessing, reconstruction and post processing, which are 3(2)ms, 13(2)ms and 13(1)ms, respectively. Relative to the acquisition time of a single frame, which is 13 · 2.1ms= 27.3ms, all steps are relatively fast. Post-processing includes removal of oversampling and a median filter. Without the median filter post processing time is 2(1)ms.

The latency measured from the perspective of the software running on the scanner matches the total latency measured within BART with a difference of approximately 5ms corresponding to the network transfer.

The end-to-end latency of 205(20)ms is several times larger than the other two latency measurements with only 28(2)ms from the BART reconstruction.

This discrepancy between end-to-end latency compared to the other measurements has multiple sources. For instance, the acquisition time itself contributes to the end-to-end latency. Furthermore, time-dependent filters such as the median filter do not affect the signal processing time too much, but have a large impact on the end-to-end latency. Without median filter, an end-to-end latency of 146(13)ms is achieved. Several other possible sources are treated in the discussion.

### 3.2 Real-Time Applications

**Figure 8:**
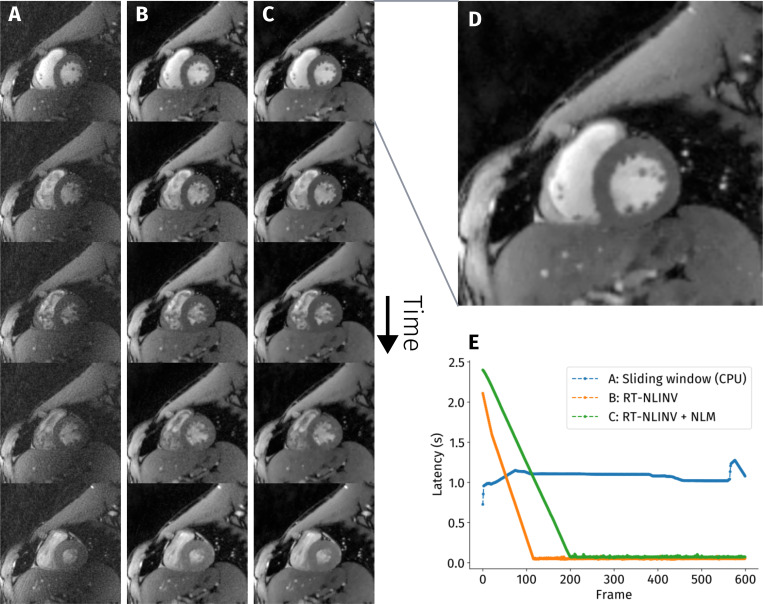
Five representative frames covering one heartbeat from a real-time acquisition of a cardiac short-axis view, reconstructed with different algorithms in a BART real-time pipeline. The first column **A** shows a sliding-window adjoint NUFFT reconstruction, whereas the other columns show the result from reconstruction with RT-NLINV. In column **C** additionally a Non-Local Means (NLM) filter is applied, and a higher number of iterations is used. Part **D** provides an enlarged view of a reconstruction with RT-NLINV + NLM filter. The graph **E** shows the numerically determined latency of all configurations. The sliding window reconstruction used standard workstation hardware without GPU acceleration.

In our experimental setup, the simple reconstruction based on the adjoint NUFFT can be run in real-time on standard hardware without use of a GPU as demonstrated by the latency graph shown in part E of Figure 8.

Iterative reconstruction methods such as RT-NLINV can be used to achieve high image quality, which can be further improved through post processing, as seen in Figure 8B-D. The initially higher, but then quickly decreasing latency in these cases is probably due to initialization of the GPU and precomputing steps.

**Figure 9:**
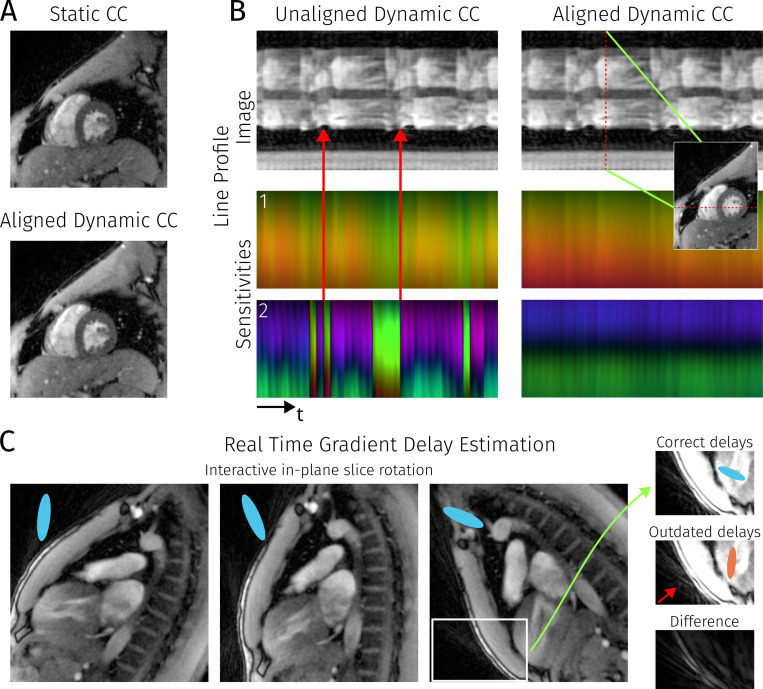
Part **A** compares two frames from a RT-MRI of the heart with different strategies for coil compression, either re-using the compressing matrix or recomputing and aligning it for every frame. The red arrows highlight abrupt changes in image magnitude which correlate with the discontinuities in the virtual coil profiles. Part **B** shows a time series from a line through the heart, with the line being shown in the small inset image. The bottom row shows the time series for the first two virtual coil profiles, the top row shows the actual images. On the left, every frame was independently compressed, on the right, compression matrices were aligned using geometric coil compression. Part **C** highlights a different aspect of preprocessing, showing three frames from an interactive real-time MRI session where the in-plane rotation was changed over time. Estimated gradient delays are visualized using ellipses shown in the top left corner of the respective images. A zoom-in on the right shows two reconstructions of the last frame, using either the current gradient delay estimation (top) or gradient delay estimations from the first frame (center), resulting in increased artefacts (red arrow).

Part A and B of Figure 9 show the effects of real-time geometric coil compression as described in Section 2.5. This is compared to the usual SVD-based coil compression with either a constant compression matrix (static cc), or a compression matrix which is updated for every frame (unaligned/aligned dynamic cc). In Figure 9A, it can be seen that noise is increased after a change of slice position, as the compression matrix is not adapted to the changed coil profiles in the new slice. Recomputing the coil compression matrix for every frame leads to problems as shown in Figure 9B: The coil profiles show distinct jumps over their time course and this then also affects the reconstructed images. These discontinuities disappear when using geometric coil compression along the time dimension.

Figure 9C focusses on gradient delays, which are shown as ellipses with principal axes proportional to the delay-induced shift in k-space in that direction. Slice orientation was approximately sagittal, meaning there is a distinct anisotropy in the gradient delays due to hardware differences between xy- and z-gradients. This is clearly visible in the elongated shape of the ellipses.

Furthermore, one can see that in-plane rotation causes the corresponding ellipse to rotate with the image, reflecting the underlying hardware gradient delays. Not updating gradient delay estimations, and thus reconstructing with a wrongly corrected trajectory, can produce increased image artefacts.

## 4 Discussion

Streaming functionality was added to the BART reconstruction software as new feature. The implementation achieves high modularity and interoperability for real-time reconstruction pipelines by relying on standard operating system utilities. Based on a set of new options, all tools included in the toolbox can be used in real-time reconstruction pipelines. Even advanced reconstruction pipelines for real-time MRI can be constructed by chaining BART commands using a scripting language. This enables a quick transition from offline prototyping of a new reconstruction method in BART to online reconstruction.

The streaming extension was tested and benchmarked in numerical experiments and using phantom and in-vivo measurements. As it is now possible to parallelize subsequent reconstruction steps in a pipeline, a speed-up can be achieved for conventional reconstruction pipelines simply by chaining BART commands and without having to call BART tools as part of a more complicated loop. Under certain circumstances, the overhead associated with streaming and looping can have a negative effect on performance, but in all tested conditions overhead was either small in absolute units, small compared to the total computation time, or small relative to the run-to-run variations. In most practical applications, the overhead should not be relevant or even be unnoticeable.

For interactive applications, the overall latency of the RT-MRI system is important. We measured numerical and end-to-end latency in a real-time setup for interventional MRI that makes use of iterative reconstruction. Here, we found that the part of the latency corresponding to the BART pipeline is only a small fraction of the overall end-to-end latency. Some part of the additional latency is explained by the median filter used in post processing that delays changes in image content by half of the filter length, and the duration of the data acquisition itself. But even taking this into account, there is still a large unexplained difference. Temporal regularization as used in RT-NLINV could be another potential source of additional end-to-end latency. However, in [42] it was shown that RT-NLINV without additional temporal filtering provides quite high temporal acuity. This suggests that the impact on the latency is probably also small. Furthermore, preliminary experiments in which we compared regular frame-wise NLINV with RT-NLINV also hint at a negligible impact of the temporal regularization on the end-to-end latency. Another probable source of end-to-end latency is image processing. As the vendor software is a black box to us, it is not clear what causes the additional latency. Possible causes include additional processing stages in the vendor software such as distortion correction, and displaying of the images, or overhead caused by storing the images in a database.

To demonstrate the flexibility of the new streaming framework, we showed examples that extend from simple NUFFT-based reconstruction which can be used even on desktop systems to advanced iterative reconstruction that achieves image quality and makes use of GPU acceleration. The advantages of dynamic coil compression and gradient delay correction in interactive real-time where shown, demonstrating how advanced functionality can be implemented in a modular pipeline. We provide a versatile reconstruction script for real-time as part of the BART toolbox that includes all the functionality described in this work. New BART functionality such as removal of phase pole artefacts [43] were also already integrated and shown to be beneficial in preliminary experiments.

Further advantages of the streaming capabilities in BART remain to be explored this includes computational aspects such as the reduction of maximal memory usage in high-dimensional reconstruction, the use of cloud computing by streaming into the cloud, or distributing the reconstruction pipeline over different systems. In the past, BART has been already integrated with the Gadgetron reconstruction framework [44–46]. It already provides tools to read and write the ISMRM Raw Data (ISMRMRD) format [47] and work is ongoing also integrate the ISMRMRD/MRD streaming protocol into BART.

## 5 Conclusion

To summarize, the streaming extension to the BART framework enables efficient reconstruction for RT-MRI applications with low latency, while retaining the full modularity of the BART framework.

## Figures and Tables

**Table 1:** Parameters of the RF-spoiled radial FLASH MRI measurements.

Parameter	Value
ADC Samples (2 × oversampling)	256
Spokes / frame	13
Matrix size	192
Flip angle	10°
TR	2.1 ms
TE	1.25 ms
FOV	256 mm
Bandwidth	1300 Hz pixel^−1^
Slice thickness	5mm

## Data Availability

In the spirit of reproducible research, the code to reproduce the results of this paper is available at https://gitlab.tugraz.at/ibi/mrirecon/papers/bart-streams (version v0.1). All reconstructions have been performed with BART, available at https://github.com/mrirecon/bart. The data used in this study is available at Zenodo 10.5281/zenodo.17671124.

## References

[1] Dietz B, Fallone BG, Wachowicz K. Nomenclature for real-time magnetic resonance imaging. Magn. Reson. Med 2019; 81:1483–1484. 30183101 10.1002/mrm.27487

[2] Buecker A, Neuerburg JM, Adam GB, Real-time MR fluoroscopy for MR-guided iliac artery stent placement. Journal of Magnetic Resonance Imaging 2000; 12:616–622. 11042645 10.1002/1522-2586(200010)12:4<616::aid-jmri15>3.0.co;2-f

[3] Ratnayaka K, Faranesh AZ, Hansen MS, Real-time MRI-guided right heart catheterization in adults using passive catheters. Eur. Heart J. 2013; 34:380–389. 22855740 10.1093/eurheartj/ehs189 PMC3561614

[4] Campbell-Washburn AE, Faranesh AZ, Lederman RJ, Hansen MS. Magnetic resonance sequences and rapid acquisition for MR-guided interventions. Magn. Reson. Imaging Clin. N. 2015; 23:669–679. 10.1016/j.mric.2015.05.006 PMC4621782 26499283

[5] Unterberg-Buchwald C, Ritter CO, Reupke V, Targeted endomyocardial biopsy guided by real-time cardiovascular magnetic resonance. J. Cardiov. Magn. Reson. 2017; 19:45. 10.1186/s12968-017-0357-3 PMC5395773 28424090

[6] Nageotte SJ, Lederman RJ, Ratnayaka K. MRI Catheterization: Ready for Broad Adoption. Pediatric Cardiology 2020; 41:503–513. 32198594 10.1007/s00246-020-02301-6 PMC7416558

[7] Neves Silva S, Aviles Verdera J, Tomi-Tricot R, Real-time fetal brain tracking for functional fetal MRI. Magn. Reson. Med. 2023; 90:2306–2320. 37465882 10.1002/mrm.29803 PMC10952752

[8] Nayak KS, Pauly JM, Nishimura DG, Hu BS. Rapid ventricular assessment using real-time interactive multislice MRI. Magn. Reson. Med. 2001; 45:371–375. 11241692 10.1002/1522-2594(200103)45:3<371::aid-mrm1048>3.0.co;2-z

[9] Narayanan S, Nayak K, Lee S, Sethy A, Byrd D. An approach to real-time magnetic resonance imaging for speech production. J. Acoust. Soc. Am. 2004; 115:1771–1776. 15101655 10.1121/1.1652588

[10] Niebergall A, Zhang S, Kunay E, Real-time MRI of speaking at a resolution of 33 ms: Undersampled radial FLASH with nonlinear inverse reconstruction. Magn. Reson. Med. 2013; 69:477–485. 22498911 10.1002/mrm.24276

[11] Rasche V, Boer RWD, Holz D, Proksa R. Continuous radial data acquisition for dynamic MRI. Magn. Reson. Med. 1995; 34:754–761. 8544697 10.1002/mrm.1910340515

[12] Uecker M, Zhang S, Voit D, Karaus A, Merboldt KD, Frahm J. Real-time MRI at a resolution of 20 ms. NMR Biomed. 2010; 23:986–994. 20799371 10.1002/nbm.1585

[13] Uecker M, Zhang S, Frahm J. Nonlinear inverse reconstruction for real-time MRI of the human heart using undersampled radial FLASH. Magn. Reson. Med. 2010; 63:1456–1462. 20512847 10.1002/mrm.22453

[14] Uecker M, Hohage T, Block KT, Frahm J. Image reconstruction by regularized nonlinear inversion-joint estimation of coil sensitivities and image content. Magn. Reson. Med. 2008; 60:674–682. 18683237 10.1002/mrm.21691

[15] Nayak KS, Lim Y, Campbell-Washburn AE, Steeden J. Real-Time Magnetic Resonance Imaging. J. Magn. Reson. Imaging 2022; 55:81–99. 33295674 10.1002/jmri.27411 PMC8435094

[16] Sorensen TS, Atkinson D, Schaeffter T, Hansen MS. Real-time reconstruction of sensitivity encoded radial magnetic resonance imaging using a graphics processing unit. IEEE Trans. Med. Imag. 2009; 28:1974–1985. 10.1109/TMI.2009.2027118 19628452

[17] Schaetz S, Uecker M. A multi-GPU programming library for real-time applications. In: Int. Conf. Alg. Arch. Parallel Process. Vol. 12. Fukuoka; 2012:114–128.

[18] Blumenthal M, Heide M, Holme C, Mrirecon/Bart: Version 0.9.00. Zenodo. 2023. doi: 10.5281/ZENODO.592960.

[19] Uecker M, Lai P, Murphy MJ, ESPIRiT—an eigenvalue approach to autocalibrating parallel MRI: where SENSE meets GRAPPA. Magn. Reson. Med. 2014; 71:990–1001. 23649942 10.1002/mrm.24751 PMC4142121

[20] Blumenthal M, Luo G, Schilling M, Holme HCM, Uecker M. Deep, deep learning with BART. Magn. Reson. Med. 2023; 89:678–693. 36254526 10.1002/mrm.29485 PMC10898647

[21] Blumenthal M, Fantinato C, Unterberg-Buchwald C, Haltmeier M, Wang X, Uecker M. Self-supervised learning for improved calibrationless radial MRI with NLINV-Net. Magn. Reson. Med. 2024. doi: 10.1002/mrm.30234. PMC12328127 39080844

[22] Message Passing Interface Forum. MPI: A Message-Passing Interface Standard Version 4.1. 2023. url: https://www.mpi-forum.org/docs/mpi-4.1/mpi41-report.pdf.

[23] Blumenthal M, Uecker M. Large-Scale High-Dimensional Image Reconstruction Via Delayed And Distributed Computing With BART. In: Proc. Intl. Soc. Mag. Reson. Med. 2025:1281.

[24] Fortran 90. Standard. Geneva, CH: International Organization for Standardization; 1991.

[25] van der Walt S, Colbert SC, Varoquaux G. The NumPy Array: A Structure for Efficient Numerical Computation. Comput. Sci. Eng. 2011; 13:22–30.

[26] Ritchie DM. The Evolution of the UNIX Time-sharing System. AT & T Bell Laboratories Technical Journal 1984; 63:1577–1593.

[27] Cerf V, Kahn R. A Protocol for Packet Network Intercommunication. IEEE Transactions on Communications 1974; 22:637–648.

[28] Lyon G. Nmap network scanning. Nmap Project; 2009.

[29] O’Sullivan JD. A fast sinc function gridding algorithm for Fourier inversion in computer tomography. IEEE Trans. Med. Imag. 1985; 4:200–207. 10.1109/TMI.1985.4307723 18243972

[30] Ramachandran G, Lakshminarayanan A. Three-dimensional reconstruction from radiographs and electron micrographs: application of convolutions instead of Fourier transforms. PNAS 1971; 68:2236–2240. 5289381 10.1073/pnas.68.9.2236 PMC389392

[31] Huang F, Vijayakumar S, Li Y, Hertel S, Duensing GR. A software channel compression technique for faster reconstruction with many channels. Magn. Reson. Imaging 2008; 26:133–141. 17573223 10.1016/j.mri.2007.04.010

[32] Zhang T, Pauly JM, Vasanawala SS, Lustig M. Coil compression for accelerated imaging with Cartesian sampling. Magn. Reson. Med. 2013; 69:571–582. 22488589 10.1002/mrm.24267 PMC3396763

[33] Schaten P, Blumenthal M, Rapp B, Uecker M. BART streams: a plug & play framework for interactive real-time MRI at the example of aligned dynamic coil compression. In: Book of Abstracts ESMRMB 2024 Online 40th Annual Scientific Meeting. 2024:719–721.

[34] Gansner ER, North SC. An open graph visualization system and its applications to software engineering. Software: Practice and Experience 2000; 30:1203–1233.

[35] Rosenzweig S, Holme HCM, Uecker M. Simple auto-calibrated gradient delay estimation from few spokes using Radial Intersections (RING). Magn. Reson. Med. 2019; 81:1898–1906. 30489652 10.1002/mrm.27506

[36] Buades A, Coll B, Morel JM. A Non-Local Algorithm for Image Denoising. In: 2005 IEEE Computer Society Conference on Computer Vision and Pattern Recognition (CVPR). Vol. 2. 2005:60–65.

[37] Zhang S, Block KT, Frahm J. Magnetic resonance imaging in real time: advances using radial FLASH. J. Magn. Reson. Imaging 2010; 31:101–109. 19938046 10.1002/jmri.21987

[38] Scholand N, Schaten P, Graf C, Rational approximation of golden angles: Accelerated reconstructions for radial MRI. Magn. Reson. Med. 2025; 93:51–66. 39250418 10.1002/mrm.30247 PMC12034029

[39] Haase A, Frahm J, Matthaei D, Hanicke W, Merboldt KD. FLASH imaging. Rapid NMR imaging using low flip-angle pulses. J. Magn. Reson. 1986; 67:258–266. 10.1016/j.jmr.2011.09.021 22152368

[40] Roeloffs V, Voit D, Frahm J. Spoiling without additional gradients: Radial FLASH MRI with randomized radiofrequency phases. Magn. Reson. Med. 2016; 75:2094–2099. 26094973 10.1002/mrm.25809

[41] Schaten P, Blumenthal M, Uecker M. BART Streams: End-to-End Latency of Real-Time MRI. In: Book of Abstracts ESMRMB 2025 Online 41st Annual Scientific Meeting. 2025:496–497.

[42] Frahm J, Schätz S, Untenberger M, On the Temporal Fidelity of Nonlinear Inverse Reconstructions for Real- Time MRI – The Motion Challenge. The Open Medical Imaging Journal 2014; 8:1–7.

[43] Blumenthal M, Uecker M. Phase-Pole-Free Images and Smooth Coil Sensitivity Maps by Regularized Nonlinear Inversion. 2025. arXiv: 2508.04685. 10.1002/mrm.70333 PMC13156459 41820227

[44] Hansen MS, Sørensen TS. Gadgetron: An open source framework for medical image reconstruction. Magn. Reson. Med. 2013; 69:1768–1776. 22791598 10.1002/mrm.24389

[45] Diakite M, Campbell-Washburn AE, Xue H. Integration of the BART Toolbox into Gadgetron Streaming Framework for Inline Cloud-Based Reconstruction. In: Proc. Intl. Soc. Mag. Reson. Med. Vol. 26. Paris; 2018:2861.

[46] Veldmann M, Ehses P, Chow K, Nielsen JF, Zaitsev M, Stöcker T. Open-source MR imaging and reconstruction workflow. Magn. Reson. Med. 2022; 88:2395–2407. 35968675 10.1002/mrm.29384 PMC10054460

[47] Inati SJ, Naegele JD, Zwart NR, ISMRM Raw data format: A proposed standard for MRI raw datasets. Magn. Reson. Med. 2016; 77:411–421. 26822475 10.1002/mrm.26089 PMC4967038

